# Evaluation of the antidiarrheal activity of the leaf extracts of *Myrtus communis* Linn (Myrtaceae) in mice model

**DOI:** 10.1186/s12906-017-1625-3

**Published:** 2017-02-10

**Authors:** Mekonnen Sisay, Ephrem Engidawork, Workineh Shibeshi

**Affiliations:** 1 School of Pharmacy, College of Health and Medical sciences, Haramaya University, Dire Dawa, Ethiopia; 2 Department of Pharmacology and clinical Pharmacy, College of Health Sciences, Addis Ababa University, Addis Ababa, Ethiopia

**Keywords:** *Myrtus communis*, Castor oil, Antidiarrheal activity, 80%ME, Solvent fractions

## Abstract

**Background:**

*Myrtus communis* L. has a folkloric repute for the management of diarrhea and dysentery in different parts of the world. However, the safety and efficacy of the leaf extract have not been scientifically validated in animal model. This study was, therefore, aimed to investigate the antidiarrheal effect of 80% methanol extract (80ME) and solvent fractions of the leaves of *Myrtus communis* L. in mice.

**Methods:**

The antidiarrheal activity of the 80ME and solvent fractions was evaluated against castor oil induced diarrheal model, charcoal meal and enteropooling tests. For the 80%ME, the test groups received 100, 200 and 400 mg/kg of the extract. In case of fractions, the test groups received various doses of fractions (200, 300, 400 mg/kg and an additional dose of 800 mg/kg for the aqueous fraction (AF)), where as negative controls received the vehicle (10 ml/kg) and positive controls received loperamide (3 mg/kg).

**Results:**

The 80ME at 200 mg/kg (*p* < 0.05) & 400 mg/kg (*p* < 0.01) as well as the chloroform fraction (CF) and methanol fraction (MF) at 400 mg/kg (*p* < 0.05) significantly delayed the onset of diarrhea. Besides, the 80ME (at all tested doses) and both of these fractions (at 300 & 400 mg/kg) significantly decreased the frequency and weight of fecal outputs. Results from the charcoal meal test revealed that the 80ME, at all doses, (*p* < 0.001) as well as the CF and MF at 300 mg/kg (*p* < 0.05) & 400 mg/kg (*p* < 0.001) produced a significant anti-motility effect. Similarly, in the entero-pooling test, the 80ME (at all tested doses) (*p* < 0.01) as well as the CF and MF (at 300 & 400 mg/kg, *p* < 0.05) produced a significant decline in the weight and volume of intestinal contents, whereas the AF revealed significant effect (*p* < 0.05) at dose of 800 mg/kg only.

**Conclusion:**

The study demonstrated that the 80ME and solvent fractions contain bioactive constituents that have antidiarrheal activity. Therefore, this study provides a scientific support for the acclaimed traditional use of *Myrtus communis* L for the treatment of diarrheal diseases.

## Background

Since the time immemorial, medicinal plants have played an invaluable role in the development of therapeutic agents. Currently, it is estimated that about 80% of people living in developing countries still rely on traditional medicine for their primary health care [1]. There are many medicinal plants that possess antidiarrheal activity with lesser side effects than the conventional drugs. Tannins, alkaloids, flavonoids and terpenoids are the major constituents that are primarily responsible for antidiarrheal activity of these medicinal herbs [2]. In Ethiopia, a range of medicinal plants have been widely used for the management of diarrhea and related gastrointestinal disorders by traditional healers [3–5]. However, the safety and therapeutic potentials of some of these medicinal plants have not been validated yet. Among them, *Myrtus communis* L. is one of the popular medicinal plants being used in the traditional medicine.

*Myrtus communis* L. (Myrtaceae) is the only species of the genus found in the Northern Hemisphere. It is an aromatic evergreen perennial shrub native to Southern Europe, North Africa and West Asia. *Myrtus*, the Greek name for Myrtle and *communis* means a common plant growing in groups [6–8]. In Ethiopia, *it* has several vernacular names such as Ades (Amharic, Guragegna, Tigregna); Haddus (hararegna), Addisaa, coddoo (Afan Oromo) [9]. It is one of the most important drugs being used in Unani system of medicine since ancient Greece. It is a well-known shrub for its therapeutic, cosmetic and food uses [8]. It has also been frequently used for various ailments like gastric ulcer, diarrhea, dysentery and rheumatism [8].

Moreover, the leaves of *Myrtus communis* L. are traditionally used for abdominal pain and diarrhea in Pakistanian and Indian traditional medicines [10], in Turkish traditional medicine (the leaves are boiled and the stock is drunk) [11] and in Iranian traditional medicine [12]. In Ethiopia, It has been used as antipyretic and sedative agent [13], anti-dandruff (bathing with crushed fresh leaves) [5], antidiarrheal and stomachic (the leaves are soaked with water overnight and the juice is taken orally in the morning) [5]. Previous in vitro study on isolated tissue preparations demonstrated that 70% methanolic extract of the leaves of *Myrtus communis* L. revealed antispasmodic, bronchodilator and vasodilator activities [14]. Besides, the essential oil of *Myrtus communis* L. (myrtle oil) also possessed significant antidiarrheal activity both in vivo and ex-vivo [15]. Therefore, extensive folkloric uses and previous studies were used as a baseline data to validate the antidiarrheal activity of the leaf extracts of *Myrtus communis* L. in mice.

## Methods

### Drugs and chemicals

All solvents used for the extraction process are of laboratory grade. Drugs and chemicals used in the study include: castor oil (Amman Pharmaceutical Industries, Jordan), activated charcoal (Acuro Organics Ltd, New Delhi, India), Loperamide (Daehwa Pharmaceuticals, Republic of Korea), distilled water (Ethiopian Pharmaceutical Manufacturing Factory, Epharm, Ethiopia), Tweens 80 (Atlas Chemical Industries Inc, India), chloroform (Hi-Media Laboratory Reagents, India), absolute methanol (Carlo Erba reagents, S.A.S., France), glacial acetic acid (BDH Laboratory Supplies Poole, England), sulfuric acid (BDH Laboratory Supplies Poole, England), ammonia(BDH Limited poole, England), hydrochloric acid(BDH Laboratory Supplies Poole, England), acetic anhydride (May and Baker LTD Dagenham, England), ferric chloride (BDH Laboratory Supplies Poole, England), Mayer’s and Dragendorff’s reagents(May and Baker LTD Dagenham, England).

### The plant material

The leaves of *Myrtus communis L.* were collected from Mersa town, North Wollo zone, Amhara region (490 km northeast of Addis Ababa) in October, 2014. The plant was authenticated by a taxonomist and a voucher specimen (number MS 002) was deposited at the National Herbarium, College of Natural and Computational Sciences, Addis Ababa University (AAU) for future reference. The leaves were washed gently and dried at room temperature under shade for 2 weeks. The dried leaves were then reduced to appropriate size using mortar and pestle.

### Experimental animals

Swiss albino mice of either sex weighing 20–30 g and aged 6–8 weeks were used for the experiment. The mice were obtained from animal house of School of Pharmacy, AAU and Ethiopian Public Health Institute (EPHI). The animals were kept in plastic cages at room temperature and on a 12 h light/dark cycle with access to pellet food and water ad libitum. The mice were acclimatized to laboratory condition for 1 week prior to the experiment. Food was withdrawn 18 h prior to the beginning of all the experiments. However, water was accessed except in entero-pooling, where both food and water were withdrawn. The care and handling was according to international guidelines for the use and maintenance of experimental animals [16, 17].

### Extraction of the plant material

#### Preparation of 80ME

Hundred fifty gram of the dried powder was weighed using a digital balance and added to an Erlenmeyer flask to which 500 ml of 80ME solvent was poured in the first round. The plant material was macerated for 72 h with occasional shaking using mini-orbital shaker (Bibby scientific limited stone Staffordshire, SI150SA, UK) at 120 rpm. The extract was filtered through double layered muslin cloth followed by Whatman No.1 filter paper (Schleicher and Schuell Micro science Gmbh, Germany). The marc was then re-macerated for a second and third time by adding another fresh solvent. The resultant filtrates were combined and concentrated using a rotary evaporator (Buchi labortechnik AG, Switzerland) under reduced pressure at 40 °C. A dark green paste was obtained and kept into a deep freezer (AFTRON AFF 545, Denmark) to solidify. The residual aqueous solvent was then removed using a lyophilizer (Operon, Korea vacuum limited, Korea). The percentage yield of 80ME was then found to be 16.33% (w/w). Finally, the extract was kept in deep freeze with an air tight container until the experiment.

### Preparation of solvent fractions

Both Soxhlet and maceration techniques were used for extraction of the plant material. The initial procedure closely resembles to that of the 80ME. However, the dried leaves were pulverized to coarse powder using mortar and pestle and then sieved to maintain uniformity of particle size. From this, 150 g dry powder was subjected to successive soxhlet extraction with solvents of increasing polarity (chloroform and absolute methanol) followed by maceration of the marc of methanol with distilled water.

In every batch, 50 g of the powdered plant material was added into the extraction thimble which was then placed into the chamber of the Soxhlet apparatus. First, 350 ml chloroform was added into the bottom flask that had been fixed with Soxhlet apparatus and heat was applied until clear liquid contents of the chamber siphoned into the solvent. The CF was then subjected to filtration with suction filter. Besides, it was concentrated using rotary evaporator under reduced pressure set at 40 °C followed by dried with oven at room temperature for 48 h. The marc (in the thimble) was collected and then dried overnight at room temperature to remove chloroform. The dried marc was re-extracted using absolute methanol using the same procedure as described for the CF except that the MF was kept for a week in an oven at room temperature. Besides, the marc of MF was then collected and dried at room temperature overnight. Finally, the dried marc of methanol was combined from every batch and macerated in an Erlenmeyer flask with distilled water and allowed to stand at room temperature for a period of 3 days in each round (total of 9 days) with occasional shaking using mini-orbital shaker. The procedure utilized for extraction of 80ME was repeated except that lyophilization instead of vaporization was used directly to concentrate the extract. After drying, the percentage yields of all fractions were determined and found to be 5.2, 13.8 and 7.2% for the CF, MF and AF, respectively. The fractions were kept in deep freeze with air tight containers till use.

### Acute oral toxicity test

According to the Organization for Economic Cooperation and Development (OECD) guidelines 2008: 425, a single female mouse was fasted for 3 h and was loaded with 2000 mg/kg of the 80ME as a single dose by oral gavage. It was then observed for any sign of toxicity within the first 24 h. Based on the results of the first mouse; another 4 female mice were recruited and fasted for 3 h. Thereafter, they were given the same dose and observed for any sign of toxicity or death in the next 14 days [18].

### Grouping and dosing

Mice were randomly assigned into five groups of six animals each for both 80ME and solvent fractions. All groups received their respective treatments using oral gavage. The first group was assigned as negative control and received a vehicle (distilled water for 80ME, MF and AF and 2% tweens-80 for the CF) at 10 ml/kg. The second group was assigned as positive control and received the standard drug, loperamide (3 mg/kg). For the 80ME, the dose levels of test groups were determined based on the acute toxicity test [18] and they are treated with 100 mg/kg, 200 mg/kg and 400 mg/kg of 80ME. Coming to the solvent fractions, however, the doses of test groups were determined based on series of pilot studies and hence test groups were treated with various doses of the fractions (200 mg/kg, 300 mg/kg and 400 mg/kg and an additional dose of 800 mg/kg for the AF). The 80ME and solvent fractions were reconstituted with the respective vehicles at appropriate concentrations. The solutions were prepared fresh on the day of the experiments.

### Determination of antidiarrheal activity

#### Castor oil (CO) induced diarrhea

The method followed by Umer et al. was used for this study [19]. Swiss albino mice of either sex were fasted for 18 h, randomly allocated to five groups of six animals each and treated as described previously. One hour after administration of the respective doses and treatments, all animals received 0.5 ml of CO. Thereafter, they were individually placed in cages where the floor was lined with white paper. During an observation period of 4 h, the onset of diarrhea, frequency of defecation and the weight of fecal output (wet and total feces in gram) were recorded for individual mouse. The percentages of diarrheal inhibition and weight of fecal output were determined according to the formulae 1–3 [20, 21].

1 $$ \%\ \boldsymbol{of}\ \boldsymbol{inhibition}\kern0.5em  = \frac{\boldsymbol{Average}\ \boldsymbol{number}\ \boldsymbol{of}\kern0.5em \boldsymbol{WFC}-\boldsymbol{Average}\ \boldsymbol{number}\ \boldsymbol{of}\ \boldsymbol{WFT}\ }{\boldsymbol{Average}\ \boldsymbol{number}\kern0.5em \boldsymbol{of}\ \boldsymbol{WFC}}\mathbf{X}\ 100 $$

Where, WFC = wet feces in the control; WFT = wet feces in the test group.

2 $$ \boldsymbol{Percentage}\ \boldsymbol{of}\kern0.5em \boldsymbol{wet}\boldsymbol{fecal}\ \boldsymbol{output} = \frac{\boldsymbol{Mean}\ \boldsymbol{weight}\ \boldsymbol{of}\ \boldsymbol{wet}\boldsymbol{feces}\ \boldsymbol{of}\ \boldsymbol{each}\ \boldsymbol{group}}{\boldsymbol{Mean}\kern0.5em \boldsymbol{weight}\ \boldsymbol{of}\ \boldsymbol{wet}\ \boldsymbol{feces}\ \boldsymbol{of}\ \boldsymbol{control}}\mathbf{X}100 $$

3 $$ \boldsymbol{Percentage}\ \boldsymbol{of}\kern0.5em \boldsymbol{total}\boldsymbol{fecal}\ \boldsymbol{output} = \frac{\boldsymbol{Mean}\ \boldsymbol{weight}\ \boldsymbol{of}\ \boldsymbol{total}\ \boldsymbol{feces}\ \boldsymbol{of}\ \boldsymbol{each}\ \boldsymbol{group}}{\boldsymbol{Mean}\kern0.5em \boldsymbol{weight}\ \boldsymbol{of}\ \boldsymbol{total}\ \boldsymbol{feces}\ \boldsymbol{of}\ \boldsymbol{control}}\mathbf{X}100 $$

### CO induced gastrointestinal motility

All mice were fasted for 18 h, divided into five groups of six animals each and treated as described earlier. An hour later, 0.5 ml CO was administered. Then, 1 ml of marker (5% activated charcoal suspension in distilled water) was administered orally 1 h after CO treatment. The animals were then sacrificed after an hour and the small intestine was dissected out from pylorus to cecum. The distance travelled by the charcoal meal from the pylorus was measured and expressed as percentage of the total length of the small intestine from the pylorus to cecum (peristaltic index) as shown in formula 4. The percentage of inhibition was then expressed using the formula 5 [22].

4 $$ \boldsymbol{Peristaltic}\ \boldsymbol{index}\left(\boldsymbol{PI}\right) = \frac{\kern0.5em \boldsymbol{distance}\ \boldsymbol{travelled}\ \boldsymbol{by}\ \boldsymbol{charcoal}\ \boldsymbol{meal}}{\boldsymbol{whole}\kern0.5em \boldsymbol{length}\ \boldsymbol{of}\ \boldsymbol{small}\ \boldsymbol{intestine}\ }\ \mathbf{X}\ 100 $$

5 $$ \%\ \boldsymbol{inhibition}\kern0.75em =\kern0.75em \frac{\mathbf{PIC}-\mathbf{PIT}\ }{\mathbf{PIC}}\ \mathbf{X}\ 100 $$

Where, PIC = peristaltic index of control; PIT = peristaltic index of test group

### CO induced enteropooling activity

Intraluminal fluid accumulation was determined using the method described by Islam et al. [23]. Mice of either sex were deprived of both food and water for 18 h and treated as described in grouping and dosing section just one hour prior to oral administration of 0.5 ml CO. An hour after CO administration, the mice were sacrificed by cervical dislocation. The abdomen of each mouse was opened; the small intestine was then taken from the pyloric sphincter to ileo-caecal junction; ligated at both ends and dissected out carefully. Each small intestine was weighed and its content was then collected by gentle milking into a graduated tube and hence the volume of intestinal contents was measured. Each intestine was reweighed and the difference between the full and the empty intestines was calculated. Eventually, the percentage inhibitions of the volume and weight of intestinal contents were determined according to the formulae 6 and 7, respectively [24].

6 $$ \mathbf{Percentage}\ \mathbf{of}\ \mathbf{inhibition}=\frac{\mathbf{MVICC}-\mathbf{MVICT}}{\mathbf{MVICC}}\ \boldsymbol{X}\ 100 $$

Where, MVICC = Mean volume of the intestinal content of the control group; MVICT = Mean volume of the intestinal content of the test group.

7 $$ \mathbf{Percentage}\ \mathbf{of}\ \mathbf{inhibition}=\frac{\mathbf{MWICC}-\mathbf{MWICT}}{\mathbf{MWICC}}\ \boldsymbol{X}\ 100 $$

Where, MWICC = Mean weight of the intestinal content of the control group; MWICT = Mean weight of the intestinal content of the test group.

### The in vivo anti-diarrheal index

The in vivo antidiarrheal index (ADI) for the 80ME, solvent fractions and standard drug were determined by the following formula [25].

$$ In\  vivo\  anti\  diarrheal\  index\ (ADI)=\sqrt[3]{Dfreq\  x\  Gmeq\  x\  Pfreq} $$

Where: Dfreq = Delay in defecation time or diarrheal onset (in % of control); Gmeq = Gut meal travel reduction (in % of control) and Pfreq = purging frequency as the number of wet stool reduction (in % of control).

### Preliminary phytochemical screening

The 80ME and solvent fractions were tested for the presence of alkaloids, flavonoids, tannins, terpenoids, steroids, glycosides and saponines using standard chemical tests [26].

### Statistical analysis

Data are expressed as mean ± standard error of the mean (SEM). The data were analyzed using SPSS version 16. Statistical significance was determined by one way analysis of variance (ANOVA) followed by Tukey post Hoc test. *P*-value of less than 0.05 was considered statistically significant. When appropriate, linear regression analysis was also applied to observe the dose dependency nature of antidiarrheal effect.

## Results

### Acute oral toxicity test

The 80ME of *Myrtus communis* leaf produced neither overt toxicity nor death during the 14 days observation period following oral administration of a single dose of 2000 mg/kg. The absence of mortality and signs of overt toxicity up to 5 times the maximum effective dose of the extract suggested that 80ME has a wider safety margin and LD_50_ value greater than 2000 mg/kg in mice.

### Effects on castor oil induced diarrheal model

In the CO-induced diarrheal model, the 80ME of *Myrtus communis* leaf significantly prolonged the onset of diarrhea and reduced the frequency and weight of fecal outputs at doses of 200 mg/kg and 400 mg/kg as compared to the control. The 100 mg/kg of the extract, however, showed a statistically significant effect on frequency of wet feces (*p* < 0.001), weight of wet (*p* < 0.001) and total (*p* < 0.05) fecal outputs. Besides, the data revealed that the percentage of diarrheal inhibitions were 42.58% (*p* < 0.01), 62.52% (*p* < 0.001), and 74.96% (*p* < 0.001) at the doses of 100 mg/kg, 200 mg/kg, and 400 mg/kg, respectively (Table 1).

**Table 1:** Antidiarrheal effects of 80ME and solvent fractions of the leaves of *Myrtus communis* Linn on castor oil induced diarrheal model in mice

Dose (mg/kg)	Onset of diarrhea (Min)	No of wet feces	Total No of feces	Average weight of wet feces (gm)	Average weight of total feces (gm)	% inhibition of defecation	% WWFO	% WTFO
Control	76.67 ± 7.99	6.67 ± 0.49	7.00 ± 0.52	0.36 ± 0.02	0.37 ± 0.02	-------	-----	----
80ME 100	109.67 ± 12.15	3.83 ± 0.70^a2b1f1^	4.50 ± 0.72^b1f1^	0.20 ± 0.02^a2b2f2^	0.21 ± 0.02^a1b1f1^	42.58	55.56	56.76
80ME 200	145.00 ± 21.77^a1^	2.50 ± 0.50^a3^	3.00 ± 0.68^a2^	0.14 ± 0.03^a3^	0.15 ± 0.03^a2^	62.52	38.89	40.54
80ME 400	173.83 ± 18.03^a2^	1.67 ± 0.49^a3^	2.67 ± 0.72^a2^	0.08 ± 0.02^a3^	0.10 ± 0.03^a3^	74.96	22.22	27.03
Loperamide 3	161.50 ± 16.93^a2^	1.83 ± 0.40^a3^	2.83 ± 0.60^a2^	0.09 ± 0.02^a3^	0.11 ± 0.02^a3^	72.56	25.00	29.73
Control	80.17 ± 4.34	6.50 ± 0.43	6.83 ± 0.54	0.35 ± 0.03	0.36 ± 0.04	-----	-----	----
CF 200	123.33 ± 23.81	4.00 ± 1.00	4.33 ± 1.08	0.20 ± 0.05	0.21 ± 0.06	38.46	57.14	58.33
CF 300	140.50 ± 19.99	3.17 ± 0.65^a1^	3.67 ± 0.76	0.16 ± 0.03^a1^	0.17 ± 0.04^a1^	51.23	45.71	47.22
CF 400	152.00 ± 21.01^a1^	2.67 ± 0.76^a1^	3.17 ± 0.83^a1^	0.13 ± 0.04^a1^	0.15 ± 0.04^a1^	58.92	37.14	41.67
Loperamide 3	165.83 ± 33.17^a1^	1.67 ± 0.76^a2^	2.33 ± 1.05^a1^	0.08 ± 0.04^a2^	0.09 ± 0.04^a2^	74.31	22.86	25.00
Control	69.33 ± 8.98	7.50 ± 1.34	8.17 ± 1.28	0.42 ± 0.05	0.45 ± 0.05	------	-----	---
MF 200	104.33 ± 6.14	5.17 ± 0.40^b1^	5.67 ± 0.49^b1^	0.29 ± 0.03^b1^	0.30 ± 0.04^b1^	31.07	69.05	66.67
MF 300	136.00 ± 29.02	3.83 ± 0.87^a1^	4.33 ± 1.05	0.22 ± 0.06^a1^	0.24 ± 0.06^a1^	48.93	52.38	53.33
MF 400	155.50 ± 26.89^a1^	2.83 ± 0.95^a1^	3.50 ± 1.23^a1^	0.14 ± 0.06^a2^	0.17 ± 0.06^a2^	62.67	33.33	37.78
Loperamide 3	166.83 ± 25.23^a1^	1.83 ± 0.70^a2^	2.33 ± 0.92^a2^	0.09 ± 0.03^a3^	0.11 ± 0.04^a3^	75.56	21.43	24.44
Control	69.33 ± 8.98	7.50 ± 1.34	8.17 ± 1.28	0.42 ± 0.05	0.45 ± 0.05	------	----	---
AF 200	70.83 ± 6.53^b2j1n1^	6.83 ± 0.70^b2i1j1m1n1^	7.67 ± 0.67^b2g1i1j1n1^	0.39 ± 0.02^b3g2i2j2n2^	0.42 ± 0.02^b3g2i1j2n2^	8.93	92.86	93.33
AF 300	77.67 ± 4.86^b1j1n1^	6.33 ± 0.72^b2j1n1^	6.67 ± 0.76^b1j1n1^	0.35 ± 0.03^b2g1i1j1n1^	0.36 ± 0.04^b2i1j1n1^	15.60	83.33	80.00
AF 400	81.00 ± 3.53^b1^	5.67 ± 0.62^b1j1n1^	6.50 ± 0.62^b1j1^	0.32 ± 0.02^b2j1n1^	0.33 ± 0.02^b1j1n1^	24.40	76.19	73.33
AF 800	134.67 ± 32.67	3.50 ± 0.99^a1^	4.33 ± 1.22^a1^	0.18 ± 0.05^a2^	0.21 ± 0.05^a2^	53.33	42.86	46.67
Loperamide 3	166.83 ± 25.23^a1^	1.83 ± 0.70^a2^	2.33 ± 0.92^a2^	0.09 ± 0.03^a3^	0.11 ± 0.04^a3^	75.56	21.43	24.44

Values are mean ± SEM (*n* = 6); analysis was performed using one way ANOVA followed by Tuckey post hoc test; ^a^ compared with control values; ^b^ compared with loperamide; ^c^ compared with 100 mg/kg; ^d^ compared with 200 mg/kg; ^e^compared with 300 mg/kg; ^f^ compared with 400 mg/kg; ^g^ compared with 800 mg/kg; ^h^ compared with CF200; ^i^ compared with CF300; ^j^ compared with CF400; ^k^compared with MF200; ^m^ compared with MF 300; ^n^compared with MF 400; ^1^ *p* < 0.05, ^2^ *p* < 0.01, ^3^ *p* < 0.001; 80ME =80% methanol extract, CF = chloroform fraction, MF = methanol fraction, AF = aqueous fraction. Controls received 10 ml/kg- distilled water (for 80ME, MF and AF) and 2% Tween-80 (for CF). WWFO = Weight of wet fecal output, WTFO = weight of total fecal output

Coming to solvent fractions, both CF and MF produced a significant delay in initiation of diarrhea at 400 mg/kg (*p* < 0.05). The percentage of diarrheal inhibitions obtained as compared to control were 51.23% (*p* < 0.05) and 58.92% (*p* < 0.05) at 300 mg/kg and 400 mg/kg CF, respectively. The CF also showed a significant reduction in weight of both wet and total fecal outputs at 300 mg/kg (*p* < 0.05) and 400 mg/kg (*p* < 0.05). Similarly, the MF significantly decreased the frequency and weight of wet feces at doses of 300 mg/kg (*p* < 0.05; p < 0.05) and 400 mg/kg (*p* < 0.05; *p* < 0.01), respectively with the highest percentage of diarrheal inhibition (62.67%, *p* <0.05) obtained at 400 mg/kg. On the contrary, the AF was devoid of any significant delay in onset of diarrhea at all tested doses as compared with control (Table 1).

There was a dose-dependent reduction in the percentage of weight of wet and total fecal outputs in 80ME and solvent fractions with 400 mg/kg of the 80ME displaying the highest effect (22.22 and 27.03%). Besides, both the CF and MF revealed a moderate reduction in the percentage of both fecal outputs with 400 mg/kg MF showing the utmost effect among the solvent fractions (Table 1).

### Effects on castor oil induced intestinal transit in mice

The 80ME significantly inhibited the intestinal transit of charcoal meal at all tested doses. The data revealed that the percentage reduction of gastrointestinal transit of charcoal was 33.54% (*p* < 0.001), 46.12% (*p* < 0.001), and 62.31% (*p* <0.001) at doses of 100 mg/kg, 200 mg/kg, and 400 mg/kg, respectively. The maximum dose of this extract showed comparable anti-motility effects to that of the standard (59.61%, *p* < 0.001).

The CF and MF also significantly inhibited the intestinal transit of charcoal meal at doses of 300 mg/kg and 400 mg/kg compared to the control with the highest inhibitory effect observed at later dose of MF (47.54%, *p* < 0.001). On the contrary, the AF showed a significant antimotility effect at the dose of 800 mg/kg (38.61%, *p* < 0.01) compared to the control (Table 2).

**Table 2:** Effects of 80ME and solvent fractions of the leaves of *Myrtus communis* L on gastrointestinal transit in mice

Dose (mg/kg)	Length of small intestine (cm)	Distance moved by the charcoal meal (cm)	Peristaltic index (%)	% inhibition
Control	56.17 ± 1.42	36.67 ± 1.94	65.09 ± 2.25	-------
80ME 100	58.33 ± 0.80	25.17 ± 2.43^a3b2f2^	43.26 ± 4.37^a3b2f2^	33.54
80ME 200	58.67 ± 1.12	20.05 ± 1.09^a3^	35.07 ± 2.27^a3^	46.12
80ME 400	58.17 ± 2.01	14.33 ± 1.65^a3^	24.53 ± 2.53^a3^	62.31
Loperamide 3	56.33 ± 1.28	14.83 ± 0.91^a3^	26.29 ± 1.34^a3^	59.61
Control	57.67 ± 1.76	36.17 ± 4.18	62.33 ± 6.06	------
CF 200	56.33 ± 1.08	25.17 ± 3.46^b1^	44.88 ± 6.39^b1^	27.99
CF 300	59.50 ± 1.09	24.00 ± 1.39^a1^	40.44 ± 2.60^a1^	35.11
CF 400	60.17 ± 1.89	20.67 ± 2.43^a2^	34.37 ± 3.91^a2^	44.86
Loperamide 3	56.83 ± 1.11	14.00 ± 1.41^a3^	24.58 ± 2.32^a3^	60.56
Control	58.50 ± 0.67	36.83 ± 3.21	62.81 ± 5.11	-------
MF 200	59.17 ± 1.38	29.17 ± 1.96^b2^	49.47 ± 3.47^b2^	21.24
MF 300	59.50 ± 1.41	24.00 ± 3.33^a1^	40.29 ± 5.48^a1^	35.85
MF 400	59.00 ± 1.59	19.17 ± 3.27^a3^	32.95 ± 5.99^a3^	47.54
Loperamide 3	56.33 ± 1.28	13.67 ± 1.52^a3^	24.20 ± 2.55^a3^	61.47
Control	58.50 ± 0.67	36.83 ± 3.21	62.81 ± 5.11	------
AF 200	56.83 ± 0.95	34.00 ± 1.83^b3g1j1n1^	59.79 ± 2.98^b3g1j1n2^	4.81
AF 300	56.83 ± 1.92	31.00 ± 2.05^b2g1^	54.91 ± 4.33^b2g1n1^	12.58
AF 400	59.17 ± 0.98	29.50 ± 3.09^b2^	49.79 ± 4.95^b2^	20.73
AF 800	56.83 ± 1.17	21.83 ± 3.55^a2^	38.56 ± 6.29^a2^	38.61
Loperamide 3	56.33 ± 1.28	13.67 ± 1.52^a3^	24.20 ± 2.55^a3^	61.47

Values are mean ± SEM (*n* = 6); analysis was performed using one way ANOVA followed by Tuckey post hoc test; ^a^ compared with control values; ^b^ compared with loperamide; ^c^ compared with 100 mg/kg; ^d^ compared with 200 mg/kg; ^e^compared with 300 mg/kg; ^f^ compared with 400 mg/kg; ^g^ compared with 800 mg/kg; ^h^ compared with CF200; ^i^ compared with CF300; ^j^ compared with CF400; ^k^compared with MF200; ^m^ compared with MF 300; ^n^ compared with MF 400; ^1^ *p* < 0.05, ^2^ *p* < 0.01, ^3^ *p* < 0.001; 80ME = 80% methanol extract, CF = chloroform fraction, MF = methanol fraction, AF = aqueous fraction. Controls received 10 ml/kg- distilled water (for 80ME, MF and AF) and 2% Tween-80 (for CF)

### Effects on castor oil induced enteropooling

In this test, the 80ME showed a significant reduction in both the average volume and weight of intestinal contents (AVIC and AWIC) at all tested doses as compared to control. The percentage inhibition of volume of intestinal contents was found to be 25.58% (*p* < 0.01), 38.37% (*p* < 0.001), and 46.51% (*p* < 0.001) at doses of 100 mg/kg, 200 mg/kg, 400 mg/kg, respectively.

The CF and MF reduced both AVIC and AWIC significantly at doses of 300 mg/kg and 400 mg/kg. Maximal inhibition of the AVIC was observed at 400 mg/kg, being 38.46% (*p* < 0.01) and 40.96% (*p* < 0.01) for CF and MF, respectively. On the contrary, the AF was devoid of any significant inhibitory effect on the AVIC and AWIC up to 400 mg/kg as compared to control (Table 3).

**Table 3:** Effects of 80ME and solvent fractions of the leaves of *Myrtus communis* L on gastrointestinal fluid accumulation in mice

Dose (mg/kg)	Volume of intestinal contents (ml)	% inhibition	Weight of intestinal contents (gm)	% inhibition
Control	0.86 ± 0.07	-------	1.12 ± 0.03	-------
80ME 100	0.64 ± 0.04^a2b1f1^	25.58	0.91 ± 0.05^a2b1f1^	18.75
80ME 200	0.53 ± 0.03^a3^	38.37	0.77 ± 0.06^a3^	31.25
80ME 400	0.46 ± 0.02^a3^	46.51	0.70 ± 0.02^a3^	37.50
Loperamide 3	0.47 ± 0.04^a3^	45.35	0.71 ± 0.03^a3^	36.61
Control	0.78 ± 0.08	--------	1.02 ± 0.07	--------
CF 200	0.61 ± 0.07^b1^	21.79	0.85 ± 0.07^b1^	16.67
CF 300	0.53 ± 0.04^a1^	32.05	0.75 ± 0.04^a1^	26.47
CF 400	0.48 ± 0.04^a2^	38.46	0.69 ± 0.03^a2^	32.35
Loperamide 3	0.43 ± 0.06^a2^	44.87	0.66 ± 0.05^a3^	35.29
Control	0.83 ± 0.06	-----	1.10 ± 0.03	-------
MF 200	0.67 ± 0.04^b1^	19.28	0.93 ± 0.07^b1^	15.45
MF 300	0.58 ± 0.03^a1^	30.12	0.81 ± 0.04^a1^	26.36
MF 400	0.49 ± 0.08^a2^	40.96	0.73 ± 0.08^a2^	33.64
Loperamide 3	0.47 ± 0.04^a3^	43.47	0.68 ± 0.02^a3^	38.18
Control	0.83 ± 0.06	------	1.10 ± 0.03	-------
AF 200	0.76 ± 0.04^b2g1j1n1^	8.43	1.02 ± 0.05^b3g2j2n1^	8.18
AF 300	0.73 ± 0.07^b1j1n1^	12.05	0.98 ± 0.07^b2g1j1^	10.91
AF 400	0.68 ± 0.07	18.07	0.93 ± 0.05^b1^	15.45
AF 800	0.55 ± 0.02^a2^	33.74	0.76 ± 0.03^a2^	30.90
Loperamide	0.47 ± 0.04^a3^	43.47	0.68 ± 0.02^a3^	38.18

Values are mean ± SEM (*n* = 6); analysis was performed using one way ANOVA followed by Tuckey post hoc test; ^a^ compared with control values; ^b^ compared with loperamide; ^c^ compared with 100 mg/kg; ^d^ compared with 200 mg/kg; ^e^compared with 300 mg/kg; ^f^ compared with 400 mg/kg; ^g^ compared with 800 mg/kg, ^h^ compared with CF200, ^i^ compared with CF300, ^j^ compared with CF400, ^k^compared with MF200, ^m^ compared with MF 300, ^n^ compared with MF 400; ^1^ *p* < 0.05, ^2^ *p* < 0.01, ^3^ *p* < 0.001; 80ME = 80% methanol extract, CF = chloroform fraction, MF = methanol fraction, AF = aqueous fraction. Controls are 10 ml/kg- distilled water (for 80ME, MF and AF) and 2% Tween-80 (for CF)

### *The* in vivo antidiarrheal index

The in vivo antidiarrheal index (ADI) was measured by considering the delay in defecation (time of onset, Dfreq), gut meal travel distance (Gmeq) and purging frequency in number of wet stools as major parameters. The greatest ADI was achieved at the dose of 400 mg/kg of 80ME (83.96%). Among the solvent fractions, MF showed the highest ADI value (71.81%) at doses of 400 mg/kg (Table 4).

**Table 4:** In vivo antidiarrheal indices of 80ME and solvent fractions of the leaves of *Myrtus communis*

Dose (mg/kg)	Delay in defecation (time of onset in min, Dfreq) (%)	Gut meal travel distance (Gmeq) (%)	Purging frequency in number of wet stools (%)	In vivo antidiarrheal index (ADI)
Control	-------	-------	-------	-------
80ME 100	43.04	33.54	42.58	39.47
80ME 200	89.12	46.12	62.52	63.58
80ME 400	126.72	62.31	74.96	83.96
Loperamide 3	110.64	59.61	72.56	78.22
Control	-------	------	------	-----
CF 200	53.84	27.99	38.46	38.69
CF 300	75.25	35.11	51.23	51.34
CF 400	89.59	44.86	58.92	61.87
Loperamide 3	106.85	60.56	74.31	78.34
Control	------	------	------	------
MF 200	50.48	21.24	31.07	32.18
MF 300	96.16	35.85	48.93	55.25
MF 400	124.29	47.54	62.67	71.81
Loperamide 3	140.63	61.47	75.56	86.76
Control	-------	-------	--------	-------
AF 200	2.16	4.81	8.93	4.53
AF 300	11.54	12.58	15.60	13.13
AF 400	16.83	20.73	24.40	20.42
AF 800	94.24	38.61	53.33	57.89
loperamide	140.63	61.47	75.56	86.76

80ME = 80% methanol extract, CF = chloroform fraction, MF = methanol fraction, AF = aqueous fraction

### Preliminary phytochemical screening

The preliminary phytochemical screening of the 80ME of *Myrtus communis* leaf revealed the presence of terpenoids, flavonoids, tannins, glycosides and saponins but alkaloids and steroids are lacking. Coming to the solvent fractions, the data revealed that alkaloids were not detected in all solvent fractions and trace amounts of steroids were detected in the CF. Besides, terpenoids and flavonoids were detected in both CF and MF. Tannins were common across all fractions. Glycosides and saponins were also observed in both MF and AF. Amongst all, the 80ME and the MF appeared to be relatively rich in secondary metabolites (Table 5).

**Table 5:** Preliminary phytochemical screening of the 80% methanol extract and solvent fractions of the leaves of *Myrtus communis* L

Constituents	Crude extract	Solvent fraction		
	80ME	Chloroform	Methanol	Aqueous
Cardiac glycosides	+	_	+	+
Flavonoids	+	+	+	-
Alkaloids	_	_	_	_
Saponins	+	_	+	+
Steroids	_	+	_	_
Tannins	+	+	+	+
Terpenoids	+	+	+	_

+ = present; − = Absent

## Discussion

Medicinal plants have been used for the treatment of various disorders including diarrhea and related gastrointestinal disorders despite the fact that their safety and efficacy profiles have not been well addressed. It is, therefore, important to properly evaluate the safety and efficacy profile of medicinal plants that are being used in traditional medicines. The need for newer, more effective, cheaper and safer antidiarrheal drugs has become a paramount concern to have safe and cost effective therapeutic alternatives [2, 27]. This study was aimed to evaluate the safety and effectiveness of *Myrtus communis* L. as antidiarrheal agent.

Hydroalcoholic solvent mixtures are generally considered to give high extraction yields, owing to their expanded polarity index [28]. In general, hydroalcoholic co-solvents such as 80% methanol seem to possess the optimum solubility characteristics for initial crude extraction. Hence, 80% methanol was used as solvent of choice in the present study for extracting the *Myrtus communis* L*.* leaf. Besides, the dry leaf powder was re-subjected for fractionation with solvents of increasing polarity to get an idea about the polarity of antidiarrheal constituents of the plant.

The acute toxicity profile of the leaf extract was determined based on OECD guideline 2008:425 [18]. On this test, the LD_50_ was found to be > 2000 mg/kg for the 80ME. Generally, if the LD_50_ value of the test chemical is more than 3 times the minimum effective dose, the substance is considered as a good candidate for further studies [29]. Since the hydroalcoholic extract had a LD_50_ value of more than three times of the minimum effective dose (100 mg/kg), it was taken as a good candidate for further studies. Beyond its role for dose determination, LD_50_ can also be used for classification of chemicals. According to WHO hazard classification systems, the 80ME of the leaves of *Myrtus communis* L*.* with LD_50_ > 2000 mg/kg is designated as ‘unlikely to be hazardous’ [30]. Hence, based on the safety profile of the this extract and prior absence of any toxicity data regarding the plant, further toxicity studies were not done on the solvent fractions.

Diarrhea occurs when there is an imbalance between the secretary and absorptive processes of gastrointestinal tract and/or an alteration of motility of intestinal smooth muscles [31]. The use of CO as diarrhea inducer has been well documented [25, 32]. When administered orally, it produces irritant laxative effect mediated by its active metabolite, ricinoleic acid released by intestinal lipases. Ricinoleic acid produces local irritation and inflammation of the intestinal mucosa, causing the release of prostaglandins that eventually increase gastrointestinal motility, net secretion of water and electrolytes [24, 33].

CO induced diarrheal model was designed to assess the potential of a test substance in its overall antidiarrheal activities. The onset of defecation, the frequency and weight of fecal outputs were determined as the main parameters. The 80ME (middle and higher doses) significantly delayed the initiation of diarrhea and reduced the number and weight of both wet and total fecal outputs with the highest effects observed at later dose. Diarrhea is characterized by fecal urgency and incontinence [34, 35]. Substances exhibiting significant antidiarrheal activity may have a potential to retard the onset of diarrhea as observed at 200 and 400 mg/kg 80ME. Even though diarrhea has been defined over time by various scientific groups and organizations in different ways, greater emphasis is given on the consistency of stools than the frequency [35]. Therefore, determination of the percentage inhibition has been based on the reduction of frequency of wet fecal outputs as a good marker of antidiarrheal activity. Diarrhea is also presented with an increase in weight of defecation [35, 36]. Accordingly, the 80ME displayed a dose-dependent reduction in weight of fecal outputs indicating the antidiarrheal potential of the 80ME in this model.

Coming to the solvent fractions, both the CF and MF (at 400 mg/kg) produced significant effects in all parameters in this model. In addition, both of these fractions significantly decreased the frequency of wet feces and weight of both wet and total stools at 300 mg/kg. Generally, these fractions had comparable antidiarrheal effects but the effects were lower than that of the 80ME. Looking at the dose dependency nature of the fractions, the MF (R^2^ = 0.994) appeared to have a steeper slope than the CF (R^2^ = 0.980). This might be attributed to qualitative and quantitative differences in bioactive constituents of these fractions. On the contrary, the aqueous fraction was devoid of any significant delay in onset of diarrhea at all tested doses. This study was in line with other studies where the CF and MF of medicinal plants reduced the frequency and weight of stools [37, 38].

Non-steroidal anti-inflammatory drugs (NSAIDs) could inhibit castor oil induced diarrhea [39]. Similarly, 80% ethanolic extracts of *Myrtus communis* L. showed potent anti-inflammatory activity in a previous study [40]. This was further supported by the fact that isolated constituents from the leaves of this plant are known to suppress the biosynthesis of eicosanoids both in vivo and in vitro [41]. Certain flavonoids, tannins and terpenoids revealed antidiarrheal activities via a multitude of mechanisms [42–44]. Most of the aforementioned secondary metabolites were screened from the leaves of this plant so far [45].

The reduction of gastrointestinal motility is one of the mechanisms by which antidiarrheal agents can act [46]. It was observed that the 80ME significantly suppressed the propulsion of charcoal marker at all tested doses. This finding suggests that this extract has the ability to influence the peristaltic movement of intestine thereby indicating the presence of an antimotility activity. Besides, both the CF and MF had comparable antispasmodic effects with the highest effect revealed at 400 mg/kg of MF (47.54%, *p* < 0.001). Previous study on isolated tissue preparations demonstrated that 70% methanolic extract of *Myrtus communis* L.leaf possessed bronchodilator, spasmolytic and vasodilator activities [14]. Therefore, it is plausible to assume that the in vivo antimotility effect of the 80ME and solvent fractions could be partly ascribed to such in vitro effects. It is in line with related studies where in vitro mechanistic studies were correlated with in vivo antimotility activities [47, 48].

The third being enteropooling model was aimed to assess the secretary components of diarrhea. In this model, the 80ME extract showed significant reduction in both AVIC and AWIC at all tested doses as compared to control. Besides, both the CF and MF showed comparable percent reduction of both AVIC and AWIC at all tested doses. On the contrary, the AF was devoid of significant inhibition of intestinal fluid accumulation up to 400 mg/kg. Mascolo et al. reported that the active metabolite, ricinoleic acid might activate the nitric oxide pathway and induce nitric oxide (NO) dependent gut secretion [49]. A growing body of evidence indicated that phytochemical constituents such as terpenoids [50] and flavonoids [51] are implicated in attenuation of NO synthesis. In contrast to the aqueous fraction, the significant antisecretory activities of the 80ME as well as the CF and MF could probably be related to the existence and hence synergistic effects of flavonoids, tannins and terpenoids.

Generally, the ADI value indicates a measure of how much effective an extract is in treating diarrhea [25]. ADI was increased with dose suggesting the dose dependency nature of the parameter. The 80ME showed highest ADI value among all extracts with corresponding doses reinforcing the notion that this extract is endowed with better antidiarrheal activity compared to the solvent fractions.

Interestingly, extensive studies revealed that the leaves of *Myrtus communis* L. have shown promising antimicrobial activities against several diarrhea causing pathogens [52–54]. Therefore, in addition to its antimotility and antisecretory effects observed in this study, its overwhelming antimicrobial properties reinforcing a notion that *Myrtus communis* L. can possibly be a good candidate for diarrheas of diverse etiologies including those with infectious component.

## Conclusion

This study revealed that the 80ME and solvent fraction of the leaves of *Myrtus communis* L. are endowed with a promising antidiarrheal activity. Therefore, this finding provides a scientific support for folkloric use of *Myrtus communis* L. as antidiarrheal agent.
